# Sweet Drinks, Sour Consequences: The Impact of Sugar-Sweetened Beverages on Sperm Health, a Narrative Review

**DOI:** 10.3390/nu17101733

**Published:** 2025-05-20

**Authors:** Winnie Khine Yi Win, Maverick Wenhao Wong, Paula Benny, Zhongwei Huang

**Affiliations:** 1 Yong Loo Lin School of Medicine, National University of Singapore, Singapore 119077, Singapore; e0771346@u.nus.edu (W.K.Y.W.); maverick.wong@u.nus.edu (M.W.W.); 2 Department of Obstetrics and Gynecology, National University Hospital Singapore, Singapore 119077, Singapore; obgv421@nus.edu.sg; 3 NUS Bia-Echo Asia Centre for Reproductive Longevity and Equality, Yong Loo Lin School of Medicine, National University of Singapore, Singapore 119077, Singapore

**Keywords:** sugar-sweetened beverages, sperm health, male fertility

## Abstract

**Introduction:** The rising global consumption of sugar-sweetened beverages (SSBs) has paralleled a concerning decline in sperm quality, raising concern about potential dietary impacts on male fertility. Sperm health parameters, including count, motility, and morphology, are critical indicators of reproductive potential and may be adversely affected by excessive sugar intake. This narrative review consolidates the current evidence on the association between SSB consumption and sperm health, highlighting potential biological mechanisms. **Methods:** A targeted literature search across PubMed, Scopus, and Google Scholar was conducted, utilising keywords “sugar-sweetened beverages”, “sperm health”, and related terms. A total of 11 eligible observational and cohort studies were selected. Studies focusing solely on animal models or unrelated dietary factors were excluded. **Results:** The primary research consistently reports a negative association between high SSB consumption and sperm parameters, including reduced count and motility, and increased DNA fragmentation. Potential mechanisms include oxidative stress, hormonal dysregulation, and metabolic dysfunction linked to obesity and insulin resistance. However, variability in study design, exposure assessment, and population demographics limits generalisability of the results. **Conclusions:** The current evidence suggests that regular SSB consumption adversely affects male reproductive health through oxidative damage and hormonal imbalances. These findings underscore the importance of public health strategies to reduce SSB intake, especially among young men of reproductive age. Further longitudinal studies with standardised methodologies, particularly in underrepresented populations such as Asian cohorts, are necessary to establish causal relationships and guide clinical recommendations.

## 1. Introduction

The global consumption of sugar-sweetened beverages (SSBs) has risen significantly, increasing by 0.68 servings per week (22.9%) between 1990 and 2018 [1]. Parallel to this trend, a concerning decline in sperm health has been observed, with average sperm concentrations dropping by over 50% from 1973 to 2018—a decline that has accelerated in recent years [2]. Infertility affects approximately 17.5% of adults globally, with male factor infertility contributing to nearly half of these cases [3].

The rising consumption of SSBs has coincided with an increased prevalence of obesity, a known contributor to reduced sperm quality. Excessive sugar intake is linked to metabolic syndrome-related conditions, including elevated triglycerides, insulin resistance, and accelerated ageing. Obesity disrupts the hypothalamic–pituitary–gonadal axis, impairing gonadotropin responses and altering the ultrastructure of ejaculated sperm [4,5]. Furthermore, SSBs promote oxidative stress by generating reactive oxygen species, which compromise sperm function. This includes damage to the sperm membrane through lipid peroxidation, mitochondrial dysfunction leading to reduced motility and viability, and DNA damage that impairs fertilisation capacity [6].

Beyond its implications for male infertility, declining semen quality has been linked to broader health outcomes. Previous studies have found that semen quality is associated with long-term morbidity; poorer semen quality is associated with a significantly higher risk of hospitalisation, particularly for cardiovascular diseases and diabetes mellitus [7]. It is therefore imperative that the negative effects of SSB consumption on sperm health be well studied and reported.

### 1.1. Sugar-Sweetened Beverages

SSBs are among the most widely consumed energy-dense beverages globally [8]. SSBs represent the largest source of added sugar in the diet; a standard 12 fluid ounce (355 mL) serving of soda delivers 35.0–37.5 g of sugar and 140–150 calories [9]. Definitions of SSBs vary, but they are generally classified as beverages containing caloric sweeteners like sucrose, high-fructose corn syrup (HFCS), or fruit juice concentrates. Regulatory definitions differ by jurisdiction; for example, New York City, USA, defines SSBs as beverages with ≥25 calories or ≥6.25 g of added sugar per 8 fluid ounces (~237 mL), while the UK threshold for SSB taxation is ≥5 g of added sugar per 100 mL [9].

SSB consumption is especially prevalent among young men [10,11]. In Singapore, the Health Promotion Board (HPB) reported in 2018 that the average person consumed 60 g (12 teaspoons) of sugar daily, with over half derived from SSBs—64% of which were pre-packaged [12]. Singapore’s Ministry of Health defines SSBs as beverages containing added or naturally occurring sugars [13].

### 1.2. Defining Sperm Health and Fecundability

The World Health Organisation (WHO) 2021 guidelines for semen analysis provide the following thresholds for normal sperm parameters [14]:

1. Volume: ≥1.4 mL
2. Total count: ≥39 million
3. Motility: ≥42%
4. Viability: ≥54%
5. Morphology: ≥4%
6. Concentration: ≥15 million/mL

Seminal fluid volume is an important component in semen analysis when investigating male factor infertility. An adequate semen volume of ejaculate is needed for the transportation of sperm into the female reproductive tract to facilitate fertilisation of the oocyte [15]. The loss of semen volume may indicate issues such as obstruction of the ejaculatory duct, retrograde ejaculation, or conditions such as congenital bilateral absence of the vas deferens (CBAVD). In CBAVD, dysplasia or the absence of seminal vesicles causes a reduction in semen volume [16].

The total sperm count is another crucial component in male factor infertility. Total sperm count is closely associated with male fecundity. Multiple studies have found a significant relationship between total sperm count and results such as time-to-pregnancy and the probability of conception. An observational study of pregnant women from four countries in Europe found that the total sperm number per ejaculate was associated with a higher probability of conception. Another study of 430 healthy couples found that the probability of conception increased linearly with the sperm concentration up to 40 million/mL [17].

Sperm motility, which describes the ability of sperm to move efficiently, is also a critical factor in male fertility. Proper motility enables sperm to traverse the female reproductive tract and penetrate the oocyte for successful fertilisation [18]. Abnormal motility patterns can indicate underlying health issues or environmental factors affecting sperm quality. For instance, exposure to oxidative stress from poor nutrition, lack of exercise, environmental toxins, or undiagnosed chronic diseases has been linked to increased sperm DNA fragmentation, which can impair motility and reduce fertility [19].

Sperm viability, which refers to the percentage of live sperm in a semen sample, is crucial in sperm health analysis as it directly impacts fertility [19]. Viable sperm are essential for successful fertilisation. Even with normal sperm count and motility, low viability can hinder fertilisation. Assessing viability also provides diagnostic insights, as reduced viability may indicate infections, toxin exposure, or systemic diseases, prompting further medical evaluation [20].

Sperm morphology refers to the size and shape of sperm cells, encompassing the head, midpiece, and tail structures [21]. Assessing sperm morphology is crucial in evaluating male fertility, as abnormalities can impair the sperm’s ability to reach and fertilise an oocyte. Deviations from normal morphology, such as structural defects, can hinder motility and the capacity to penetrate the ovum. Conditions like teratozoospermia, characterised by a high percentage of abnormally shaped sperm, are associated with reduced fertility [22].

Fecundability is the probability of achieving a clinically recognised pregnancy within one menstrual cycle among couples not pregnant in the previous cycle [23].

### 1.3. Knowledge Gaps

While the links between SSB consumption and chronic diseases are well established, research on the specific relationship between SSBs and male infertility remains limited. A deeper understanding of this association could inform lifestyle interventions for couples seeking to conceive, allowing clinicians to work with their patients to devise strategies and interventions to reduce SSB consumption for reproductive health as well as to maintain metabolic health.

### 1.4. Aims of This Paper

This paper reviews the existing literature on SSB consumption and male factor infertility. By summarising current findings, we aim to highlight areas for further research and provide clinicians with evidence-based recommendations to guide preconception and the optimisation of reproductive health and overall health and well-being in men.

## 2. Methods

### 2.1. Inclusion and Exclusion Criteria

To ensure the relevance and quality of the studies included in this narrative review, specific inclusion and exclusion criteria were applied during the selection process. Studies were eligible for inclusion if they were peer-reviewed articles that investigated the relationship between SSBs and sperm health parameters, as described in an earlier section. We included research focusing on human male participants of reproductive age (18–50 years of age) to ensure findings were directly applicable to the target population. Only studies published in English within the time frame of 2000–2024 were considered, as this period was deemed reflective of contemporary dietary trends and research advancements.

Conversely, studies were excluded if they focused solely on animal models or in vitro experiments without human data, as the findings from these studies are less translatable to public health recommendations. Primary research studies investigating dietary factors without including SSBs, or studies with poorly defined methodology and insufficient data on sperm health outcomes, were excluded to maintain the focus of this review. When considering previous review articles, studies that did not have SSBs as a study variable were also excluded, and these reviews did not include the selected published primary research papers. Conference abstracts, unpublished data, and non-peer-reviewed articles were also excluded to ensure the reliability and quality of the evidence. Furthermore, studies in languages other than English were not considered, as translation resources were unavailable.

### 2.2. Confounding Factors

During the screening process, attention was paid to whether studies considered other major lifestyle and environmental confounders. Studies were prioritised if they adjusted for key factors such as smoking status, alcohol intake, occupational exposure to environmental toxins, recreational drug use, body mass index (BMI), and levels of physical activity. Studies that did not account for these potential confounders were critically evaluated for risk of bias during the synthesis and interpretation of results. However, exclusion was not strictly based on the presence or absence of adjustment given the limited availability of studies on this topic.

### 2.3. Literature Search and Screening of Studies

A literature search was carried out from 11 October 2024 to 14 December 2024 across multiple databases such as PubMed, Google Scholar and ScienceDirect using search terms related to sugar-sweetened beverages and sperm health. Specific search terms included “sugar-sweetened beverages”, “soft drinks”, “energy drinks”, “soda”, “sperm parameters”, “sperm count”, “sperm motility”, and “semen analysis”.

The initial search yielded 40 results. Duplicates were excluded and the articles were screened. Titles and abstracts were screened for relevance based on the defined inclusion and exclusion criteria, and studies that did not meet the inclusion criteria were excluded. Full-text reviews of the remaining articles were then conducted, resulting in a final selection of 11 studies which included primary research articles such as observational and cohort studies.

To minimise reporting bias, review articles that included overlapping primary studies already selected for this narrative review were excluded from the final analysis. While these reviews offered valuable background insights, our synthesis focused exclusively on independently appraised primary studies to avoid the duplication of evidence.

## 3. Results

### 3.1. General Trends in the Literature

Analysis of the literature regarding the impact of SSBs on male reproductive health, particularly sperm quality, reveals consistent trends regarding the negative associations between consumption and various sperm parameters, as shown in Table 1. Key findings demonstrated that regular SSB intake, especially in high consumption (i.e., ≥7 drinks per week, or equivalent to an estimate of 245.0–262.5 g of sugar (based on an assumption of 35.0–37.5 g of sugar per serving in a typical 12 fl oz (355 mL) of soda) [9]), correlates with a significant decline in sperm concentration and motility. Several studies reflected evidence of dose–response relationships, where higher SSB intake exacerbates negative outcomes. Hormonal disruptions through reduced inhibin-B/follicular stimulating hormone (FSH) ratios reflect possible underlying mechanisms correlating the impact of increased oxidative stress and metabolic dysfunction on sperm health.

While most of the research supports our hypothesis that increased SSB intake is correlated with a decline in sperm health, inconsistencies were noted across findings. For instance, some studies reported minimal impact on parameters such as volume or motility, and the results occasionally differed across demographic groups.

An evaluation of the study populations may help contextualise these inconsistencies. Most of the included cohorts comprised generally healthy young men, including university students, military conscripts, and individuals planning conception. The majority of the study cohorts were young men living in urban communities. Their average BMIs were typically within the normal range, and several studies adjusted for key lifestyle factors such as smoking, alcohol consumption, and physical activity. However, broader exposome factors such as sleep quality, environmental pollutant exposure, dietary antioxidant intake, and supplement use were rarely assessed or adjusted for. Only one study incorporated adjustments for both antioxidant intake and physical activity.

### 3.2. SSBs and Sperm Parameters

(I) *Semen Volume*

Joseph et al. [34] found that a higher SSB intake was inversely associated with semen volume. Men who drank ≥7 SSBs a week yielded a 6% lower semen volume than men who did not drink SSBs at all. However, the confidence interval ranged between −13% to 0%, suggesting that the reduction was not statistically significant, although a general trend towards lower semen volumes was noted. The specific intake of subtypes of SSBs, such as sugar-sweetened soda, was not significantly associated with a decrease in semen volume (%D = 0, 95% CI: −5, 5).

Yang et al. [27] found that Coca Cola consumption showed an association with a 4.1% decrease in semen volume for 1–2 bottles/week and a 12.5% decrease for ≥three bottles/week (*p* < 0.01).

Similarly, Jensen et al. [24], in their study on caffeine (including Coca Cola which is an SSB) and its impact on semen quality, found that compared to non-drinkers, the semen volume significantly decreased in men who drank more than 14 0.5-litre bottles of Coca Cola a week. When Coca Cola consumption was treated as a continuous variable, logistic regression with semen volume as an outcome found that there was an inverse association between Coca Cola consumption and semen volume (β = −0.08, CI: −0.16, −0.01).

There is therefore some evidence showing that higher SSB consumption is linked to lower semen volume, leading to reduced sperm health.

(II) *Sperm Concentration*

Liu et al. [26] found that the mean sperm concentration of individuals with intake of “highly sweet snacks & sugar-sweetened drinks” decreased significantly from 55.741 million/mL in those consuming ≤ three servings/week to 50.762 million/mL in those consuming ≥ six servings/week (*p* = 0.001). Men who consumed more SSBs had 1.289–1.358 fold higher odds of having an abnormal sperm concentration (<15 million/mL) as their intake of SSBs increased from ≤ three servings/week, four servings/week, and five servings/week to ≥ six servings/week (95% CI: 1.027, 1.618; 95% CI: 1.023, 1.681; 95% CI: 1.070, 1.723).

In another study by Nassan et al. [30], multivariable-adjusted analyses showed that men in the highest category of SSB intake (1.1 servings (~200 mL)/day) had a 13.2 million/mL lower median sperm concentration (95%CI: −21.0, −5.5) than non-consumers.

Efrat et al. [31] reported that the mean sperm concentrations in men who consumed a median of 0.2 servings/day of SSBs were 19% lower than in non-consumers (35 million/mL vs. 42 million/mL) (*p* = 0.026). The mean sperm concentration of men who consumed a median of one serving/day of SSBs was 20% lower compared to non-consumers (34 million/mL vs. 42 million/mL).

Joseph et al. [34] found that men who consumed ≥ seven SSBs a week had a 22% reduction in sperm concentration compared to non-consumers (95% CI: −38, 0). Although the upper bound of the confidence interval is at 0, there is a general trend towards a decrease in sperm concentration. SSB intake was also associated with a higher risk of low sperm concentration, with an adjusted risk ratio of 1.89 (95% CI: 1.11, 3.21). When results were stratified by the BMI, this inverse association between SSB intake and a lower sperm concentration persisted in males with a BMI of ≥ 25 kg/m^2^, suggesting a significant impact of sugar on males with a higher BMI.

Similarly, Jensen et al. [24] reported a negative association between Coca Cola consumption and sperm concentration. Men who drank 0, 1–7, 8–14, and >14 0.5 L bottles of Coca Cola had adjusted sperm concentrations (million/mL) of 56 (95% CI: 50, 64), 47 (95% CI: 44, 51), 49 (95% CI: 43, 57), and 40 (95% CI: 32, 51), respectively. Compared to men who did not consume Coca Cola, every increase in one 0.5 L bottle of Coca Cola consumed caused a 7% decrease in sperm concentration (β = −7.0, CI: −12.2, −1.69).

Therefore, there is strong evidence that increased SSB intake is linked to lower sperm concentration, with multiple studies across various cohorts reporting statistically significant results.

(III) *Total Sperm Count*

Nassan et al. [30] reported that the total sperm count of high SSB consumers (1.1 servings (~200 mL)/day) was lower by 28 million compared to non-consumers (95% CI: −48, −9).

Efrat et al. [31] found that compared to non-consumers, the mean total sperm count in men who consumed a median of 0.2 servings/day of SSBs had a 29% lower total sperm count (TSC) (*p* = 0.015), while men who consumed a median of one serving/day of SSBs had a 17% lower TSC (*p* = 0.031).

Joseph et al. [34] found that a higher SSB intake was associated with a lower total sperm count. Participants who consumed ≥ seven SSBs a week had a TSC that was 22% lower (95% CI: −38, −2) than non-consumers. Sugar-sweetened soda showed a particularly strong association with a decreased TSC—an increase in one sugar-sweetened soda a day decreased the TSC by 11% (CI: −22, 1). Men who consumed ≥ seven SSBs a week were 1.75 times more likely to have a low TSC compared to non-consumers (95% CI: 0.92, 3.33). Although the lower bound of the CI is slightly lower than one, there is a general trend towards increased risk. An additional SSB drink/day was linked to a 12% decrease in the TSC (95% CI: −22, −1). The negative association between SSB intake and the TSC was more pronounced in men with a higher BMI (≥25 kg/m^2^). SSB intake was less strongly associated with a decreased TSC in men with a lower BMI (<25 kg/m^2^), showing a 1% decrease with an additional SSB drink/day. This suggests a possible influence of body weight on the impact of SSBs on the TSC.

Similarly, Jensen et al. [24] reported a negative association between Coca Cola consumption and the total sperm count. Men who drank 0, 1–7, 8–14, and >14 0.5 L bottles of Coca Cola had total sperm counts (million) of 181 (95% CI: 156, 210), 144 (95% CI: 132, 157), 153 (95% CI: 129, 182), and 121 (95% CI: 92, 160). Compared to men who did not consume Coca Cola, every increase in one 0.5 L bottle of Coca Cola consumed caused a 9.5% decrease in the total sperm count (β = −9.5, CI: −15.2, −3.44).

Overall, the total sperm count has also been shown to be negatively affected by the consumption of SSBs.

(IV) *Sperm Motility*

Joseph et al. [34] found that increased SSB consumption was negatively associated with sperm motility, although the impact is relatively modest and not statistically significant. The negative association of SSBs with sperm motility was less pronounced compared to other parameters like the sperm concentration or total sperm count. The consumption of ≥ seven SSBs a week was associated with 4% lower sperm motility (95% CI: −10, 2), though the reduction was not statistically significant. Relative risk for low sperm motility when consuming more than seven SSBs a week was 1.23 (95% CI: 0.87, 1.75), suggesting a 23% increased risk for low sperm motility in the high SSB consumer group. Different subtypes of SSBs showed no strong evidence of effect on sperm motility, and there was no major difference in the impact of SSBs on sperm motility between high and low BMI groups.

Chiu et al. [25] found that higher SSB consumption is inversely related to sperm motility. Men in the highest quartile of intake (≥1.3 servings/day) had 6.3% lower sperm motility (95% CI: 1.0, 11.6) than men in the lower quartiles of SSB intake. Progressive motility was also affected; men in the highest quartile had 9.8% lower sperm motility than men in the lowest quartile. No difference was found in the effect of specific SSBs (carbonated SSBs with/without caffeine, non-carbonated SSBs) on sperm motility. Adjustment for BMI showed that SSBs were inversely related to sperm progressive motility in lean men, but the effect was not observedin overweight or obese men, collectively defined as BMI ≥ 25 kg/m^2^. The authors hypothesised that the weaker effect of SSBs on sperm health in overweight or obese men likely results from the stronger baseline negative impact of obesity on semen quality, which masks the smaller effect of SSBs.

Although there is some evidence that SSB consumption negatively impacts sperm motility, the evidence is weaker, with some statistically insignificant results. More research on SSBs and sperm motility may be beneficial to accurately assess the impact on sperm motility.

(V) *Sperm Morphology*

Liu et al. [26] found that the percentage of sperm with normal morphology decreased with a greater intake of “high sweet snacks & sugar-sweetened drinks”, from 67.509% in those consuming ≤ three servings/week to 66.688% in those consuming ≥ six servings/week (adjusted *p* = 0.002). Similarly, Jensen et al. [24] reported an inverse relationship between Coca Cola consumption and sperm morphology. Compared to men who did not consume Coca Cola, every increase in one 0.5 L bottle of Coca Cola consumed caused a 0.5% decrease in the percentage of morphologically normal sperm (β = −0.5, CI: −0.91, −0.09).

However, contrasting findings were reported by Kiwitt-Cárdenas et al. [32], who found that a higher SSB intake was associated with a significantly higher percentage of morphologically normal sperm (an adjusted increase of 37.2%, *p*-trend = 0.047).

Overall, while some studies suggest that a higher SSB intake is associated with a modest decrease in the percentage of morphologically normal sperm, conflicting data have been found, indicating a potential positive association, highlighting inconsistencies that warrant further investigation.

### 3.3. Association Between SSB and Hormone Levels

Chiu et al. [25] found that SSB intake was unrelated to most reproductive hormones, although there was a slight decrease in FSH from 2.7 IU/L in the lowest quartile of SSB consumers (<0.2 servings/day) to 2.2 IU/L in the highest quartile of SSB consumers (>1.3 servings/day). However, this finding was of borderline statistical significance (*p* = 0.07).

Nassan et al. [30] showed that men consuming a median of 1.1 servings (~220 mL) a day of SSBs had 12 pg/mL lower serum inhibin-B compared to non-consumers (95% CI: −21, −4). The inhibin-B/FSH ratio also decreased by nine when comparing the two groups of men (95% CI: −18, 0).

Additionally, Kiwitt-Cárdenas et al. [32] observed a trend toward higher serum oestradiol concentrations with increased SSB intake among healthy young men, although individual quartile comparisons were not statistically significant. The biological implications of modest oestradiol elevation in the context of SSB consumption remain unclear and warrant further investigation.

Although some evidence links SSBs with a reduction in hormones such as inhibin-B, it is worthwhile noting that a study by Jankowska et al. [35] showed that while lower inhibin-B is associated with a lower sperm count, some couples in their study achieved pregnancy even with low inhibin B levels (lowest inhibin B levels of 45 pg/mL for spontaneous pregnancy, 78 pg/mL for intrauterine insemination, and 34 pg/mL for in vitro fertilisation). Conversely, pregnancies were not achieved for some couples despite normal FSH or inhibin B levels. Therefore, it is proposed that fertility is determined not only by hormone levels or even sperm parameters, but also by couple-related subfertility factors.

### 3.4. Association Between SSB and Fecundability

Wesselink et al. [28], in their study on caffeine and its association with fecundability, found that male intake of caffeinated soda was negatively associated with fecundability ratios. Compared to non-consumers, one can of caffeinated soda a day was associated with a reduced fecundability ratio of 0.77 (95% CI: 0.56, 1.05), while consuming ≥ two cans of caffeinated soda a day was associated with a further reduced fecundability ratio of 0.72 (95% CI: 0.46, 1.11). The consumption of ≥one energy drink(s) a day was also associated with a substantial reduction in fecundability, and energy drink consumers had a fecundability ratio of 0.46 (95% CI: 0.21, 0.98) when compared to non-consumers.

Hatch et al. [29] reported that a male intake of SSBs was also associated with a reduction in fecundability ratios. Compared to non-consumers, an intake of 2–6 servings/week of SSBs yielded a fecundability ratio of 0.85 (95% CI: 0.71, 1.01). The intake of ≥seven servings a week of SSBs caused a further reduction, with a statistically significant decrease in the fecundability ratio of 0.78 (95% CI: 0.63, 0.96). Analysis by subgroups of SSBs showed that energy drinks were the most strongly associated with reduced fecundability: ≥seven servings a week of energy drinks yielded a fecundability ratio of 0.42 (95% CI: 0.20, 0.90), while ≥seven servings a week of sugar-sweetened sodas yielded a fecundability ratio of 0.67 (95% CI: 0.51, 0.89). In comparison, diet sodas with no sugar saw no significant impact on fecundability, with a fecundability ratio of 0.96 (95% CI: 0.72, 1.22).

While there is some evidence to suggest a general trend towards a reduction in the fecundability ratio with increased SSB intake, there are still results in the studies that are not statistically significant, suggesting that the association between SSBs and the fecundability ratio is still not clear. Further research on SSBs and their relationship with fecundability ratios will be needed to provide stronger evidence.

### 3.5. Inconclusive Findings

While most studies highlighted in this review concluded some degree of impact of SSBs on various sperm parameters, Meldgaard et al. [33] found that neither SSBs nor artificially sweetened beverages had a significant impact on sperm parameters. Compared to participants who consumed SSBs less than once a week, participants who consumed SSBs everyday had adjusted ratios of 0.98 (95% CI: 0.81, 1.18) for semen volume, 0.99 (95% CI: 0.69, 1.42) for sperm concentration, 0.95 (95% CI: 0.87, 1.03) for total motility, 0.92 (95% CI: 0.68, 1.25) for sperm morphology, and 0.99 (95% CI: 0.67, 1.46) for total sperm count. Analysis of participants consuming artificially sweetened beverages also yielded similarly statistically insignificant results. The study team hypothesised that the young age (18) of the participants selected for the study could have contributed to the inconclusive results, as other studies included participants of a wider age range.

## 4. Discussion

### 4.1. Impact of SSBs on Sperm Health

The collective evidence from observational and cohort studies underscores the negative associations between SSB consumption and various parameters of sperm health, including declines in sperm concentration, motility, and morphology. Oxidative stress resulting from excessive sugar intake appears to play a significant role in these adverse effects by causing lipid peroxidation of sperm membranes and impairing mitochondrial function, ultimately impairing sperm motility and viability [6].

Additionally, metabolic disruptions induced by SSBs, such as insulin resistance, further exacerbate oxidative damage and hormonal imbalances, impairing spermatogenesis through alterations in inhibin B/FSH ratios. Studies that adjust for variables like BMI reveal a stronger negative impact among individuals with a higher BMI, suggesting that metabolic health modulates the effects of SSBs [25,34].

### 4.2. Mechanisms Underlying Sperm Damage

(I) *Oxidative Stress and DNA damage*

Oxidative stress, characterised by the excessive production of reactive oxygen species (ROS), is a major mechanism by which SSBs impair sperm function. ROS generation leads to lipid peroxidation of sperm membranes, mitochondrial damage, and impaired motility. Furthermore, oxidative stress contributes to sperm DNA fragmentation (base mismatch, base modification, DNA adducts, DNA crosslinks, pyrimidine dimers, single-strand breaks, and double-strand breaks), which can affect sperm quality and compromise reproductive outcomes. These damages are commonly assessed using molecular assays such as the sperm chromatin structure assay (SCSA), TUNEL (terminal deoxynucleotidyl transferase dUTP nick end labelling), sperm chromatin dispersion (SCD), and the comet assay (single-cell gel electrophoresis) [36,37].

Beyond direct reproductive toxicity, SSB-induced oxidative stress has systemic vascular effects. Loader et al. demonstrated that acute SSB consumption induces transient hyperglycaemia, increases ROS production, and impairs endothelial function in both microvascular and macrovascular circulations [38]. Importantly, vascular dysfunction was reversible with antioxidant therapy (*N*-acetylcysteine and apocynin), highlighting oxidative stress as a pivotal mediator [38]. These findings reinforce the biological plausibility that SSB-induced oxidative stress can have widespread cellular impacts, including on highly ROS-sensitive cells such as spermatozoa.

Chronic oxidative stress may also contribute to accelerated cellular ageing. Leung et al. found that regular sugar-sweetened soda consumption was associated with shorter leukocyte telomere length in healthy adults, suggesting that SSBs may promote systemic oxidative damage and premature biological ageing [39]. As telomere shortening is linked to increased risk of metabolic syndrome, diabetes, cardiovascular disease, and potentially impaired reproductive function, these findings underscore the broader biological consequences of sustained high-sugar intake and oxidative burden [39].

Antioxidant supplementation, including vitamins C and E, glutathione, and coenzyme Q10, has shown promise in mitigating oxidative stress-induced damage, suggesting potential preventive strategies alongside efforts to reduce environmental and dietary oxidative exposures. Several studies report that oral antioxidant supplementation over 2–3 months can significantly reduce DNA fragmentation in men with idiopathic infertility or known oxidative stress, particularly when initial DNA fragmentation is high (≥20%) [40,41,42,43]. Although a variability in response has been reported, studies have found that those with higher initial DNA fragmentation often experience the most significant improvements [40,41]. Careful patient selection and clinical evaluation are essential before initiating routine antioxidant supplementation, and further research is needed to establish standardised protocols and optimal formulations. A balanced, antioxidant-rich diet remains a safer and more sustainable long-term strategy. The synergistic effects of a broad range of dietary antioxidants are thought to be more effective and less risky than high-dose supplementation, as excessive antioxidant intake may paradoxically impair sperm function [44,45].

(II) *Hormonal Disruption*

SSBs may also impair spermatogenesis through hormonal pathways. Pituitary-derived FSH provides indirect structural and metabolic support to the development of spermatogonia into mature spermatids via its membrane-bound receptor in Sertoli cells [46]. Inhibin-B, a testicular peptide that is secreted by Sertoli cells, negatively regulates FSH levels, which are correlated with sperm count and testicular volume [47]. Lower levels of inhibin-B point towards a lower sperm count and less efficient spermatogenesis. Nassan et al. [30] found that high SSB intake was associated with significantly lower serum inhibin-B levels and a reduced inhibin-B/FSH ratio, which are markers of Sertoli cell function. The absence of compensatory increases in FSH, even when lower sperm counts were detected, suggests that SSBs may also influence hypothalamic–pituitary signalling, further impairing spermatogenesis.

In addition to inhibin-B and FSH changes, Kiwitt-Cárdenas et al. [32] observed a trend toward elevated oestradiol levels with higher SSB consumption. Although the clinical significance of modest oestradiol increases remains uncertain, these findings suggest that SSB intake may impact the reproductive hormonal milieu beyond inhibin-B and FSH. Future studies incorporating comprehensive hormonal panels could provide more detailed insights into endocrine modulation by dietary factors.

(III) *Environmental Pollutants and Additional Oxidative Insults*

In addition to dietary factors, environmental pollutants represent a significant component of the exposome and are a major contributor to systemic oxidative stress that may impair male fertility. Recent studies demonstrate that individuals living in high environmental impact areas exhibit elevated bisphenol A (BPA) levels in blood and reproductive fluids, even after accounting for dietary intake [48]. BPA, a known endocrine disruptor, bioaccumulates through airborne exposure, promoting oxidative DNA damage and mitochondrial dysfunction. Although studies like Raimondo et al. [48] primarily focused on female reproduction, similar oxidative pathways are believed to impair sperm quality through shared mechanisms affecting gamete integrity.

However, few studies in this review comprehensively accounted for environmental exposures or associated oxidative burden from pollutants. The exposome refers to the totality of lifestyle and environmental exposures, including diet, pollution, sleep, physical activity, and other modifiable factors that can influence health outcomes. Most of the 11 studies reviewed did not measure or adjust for these variables, which limits the ability to determine whether the observed associations with SSB intake were due to direct biological effects or confounding. Only one study (Kiwitt-Cárdenas et al. [32]) adjusted for both antioxidant and physical activity, which are known modulators of oxidative stress. The absence of consistent exposome profiling may have contributed to variability in results and highlights the need for more comprehensive environmental and lifestyle assessments in future research.

### 4.3. Consequences on Fertility Outcomes

(I) *Sperm Concentration and Count*

Evidence from multiple studies [26,30,31] consistently reported an inverse relationship between SSB intake and sperm concentration. These effects are often amplified in individuals with a higher BMI, suggesting that metabolic dysfunction linked to obesity exacerbates the detrimental impact of SSBs on spermatogenesis.

(II) *Sperm Motility and Morphology*

Chiu et al. [25] and Joseph et al. [34] reported a dose–response relationship, where increased SSB consumption correlated with reduced sperm motility and abnormal morphology. However, these findings show modest statistical significance, reflecting variability in study designs and populations.

A higher SSB intake has been linked to reduced motility, including total and progressive motility, which compromises the sperm’s ability to navigate towards an ovum for fertilisation. Similarly, studies have shown a decrease in the percentage of normal sperm morphology with increased SSB intake. The effects of SSBs on sperm motility and morphology are likely due to oxidative damage, as illustrated in the paragraphs above.

While most studies observed detrimental effects of SSB intake on sperm morphology, including reductions in the percentage of morphologically normal sperm, Kiwitt-Cárdenas et al. [32] reported a contrasting finding. In their cohort of healthy young Spanish men, a higher SSB intake was associated with a significantly higher percentage of morphologically normal sperm. The biological mechanisms underlying this unexpected association remain unclear. It is possible that unmeasured confounding factors, reverse causation, or population-specific characteristics may have contributed to this finding. Further longitudinal studies are needed to clarify the directionality and causality of this relationship.

(III) *Fecundability and Clinical Outcomes*

In their paper investigating caffeine, Wesselink et al. [28] did not find caffeine as a cause of reduced fecundability, as caffeinated coffee, black tea, and green tea were not associated with reduced fecundability ratios. However, caffeinated soda and energy drinks yielded significant reductions in fecundability ratios. It is therefore reasonable to infer that caffeinated soda and energy drinks caused a reduction in fecundability due to confounders, and it is possible that it was sugar in these beverages that affected fecundability.

Hatch et al. [29] reported that an increase in SSB intake was associated with lower fecundability ratios. Most notably, energy drinks were most strongly associated with lower fecundability ratios. Other than the increased sugar content, energy drinks also contain other biologically active compounds such as caffeine and carnitine. Caffeine is a natural psychoactive chemical that has been found to negatively affect male reproductive function, postulated to be through sperm DNA damage [49]. On the contrary, L-Carnitine, a naturally occurring amino acid, has been previously shown to positively impact male fertility, particularly sperm motility, even at low doses [50]. Ultimately, the presence of stimulants in SSBs may exert confounding effects on sperm parameters, and more research on the effect of stimulants in energy drinks could further existing knowledge on factors that affect sperm health.

### 4.4. Artificial Sweeteners

Two papers in this review studied the effects of artificial sweeteners on sperm health. Hatch et al. [29] found that diet sodas containing artificial sweeteners without natural sugars had an insignificant impact on fecundability. Meldgaard et al. also reported that artificially sweetened beverages had statistically insignificant results on semen volume and total sperm count, as well as sperm concentration, motility, and morphology.

While existing research has noted the minimal impact of artificial sweeteners on sperm health, the literature is scarce and may not accurately reflect the actual impact of artificial sweeteners. It is also important to note that “artificially-sweetened beverages” is a general category that does not stratify the different types of artificial sweeteners, such as aspartame, cyclamate, and sucralose. Further research into the different artificial sweeteners present in sweetened beverages and their individual impact on sperm health may yield more insightful information.

### 4.5. Subgroup Variability

Demographic factors and underlying metabolic conditions appear to modulate the impact of SSBs on sperm parameters. Studies such as those by Joseph et al. [34] and Liu et al. [26] observed stronger negative associations in individuals with a higher BMI, possibly due to the synergistic effects of obesity and high sugar intake on metabolic dysfunction. Conversely, Chiu et al. [25] noted a greater impact on motility in lean men, which could indicate a potential protective effect of a higher BMI against some oxidative insults. As such, there is currently inconclusive evidence on the effect of the BMI on sperm parameters. Future research into BMI subgroup variability will allow better insight into BMI effects and guide clinicians in their practice.

However, while BMI serves as a general marker of body weight, caution should be taken in interpreting BMI and its effects on sperm parameters, as it is unable to account for an individual’s body composition of bone, muscle, and fat.

### 4.6. Limitations and Knowledge Gaps

While evidence generally supports the hypothesis of a negative association between SSBs and sperm health, several limitations must be acknowledged.

Firstly, most studies included in this review were cross-sectional in design, which restricts the ability to infer causality between SSB intake and changes in sperm parameters. Longitudinal studies are needed to better establish temporal relationships.

Secondly, exposure to SSBs was typically assessed using self-reported dietary questionnaires, which may be prone to recall bias and measurement error. The misclassification of SSB intake could have led to the under- or overestimation of associations.

Thirdly, while efforts were made to prioritise studies adjusting for key lifestyle factors such as smoking status, alcohol consumption, physical activity, and BMI, most studies did not fully account for the broader exposome. Important exposures such as sleep quality, environmental pollutants, dietary antioxidant intake, and supplement use were rarely assessed or adjusted for. The underlying health status and antioxidant capacity may influence oxidative stress levels and sperm health. Without comprehensive exposome profiling, residual confounding is likely to be present. This limitation is particularly relevant for studies exploring oxidative stress-mediated mechanisms, where participants’ baseline antioxidant defence systems could have modified their vulnerability to damage. The absence of these variables may partly explain inconsistencies observed between studies and limit the ability to attribute adverse outcomes solely to SSB intake.

Fourthly, although semen analysis remains a cornerstone of male fertility evaluation, it may not fully capture the underlying functional impairments of sperm, such as oxidative DNA damage, capacitation defects, or acrosomal dysfunction. While the examination of semen parameters can provide a general overview of sperm health, additional molecular assessments (hemizona assay/sperm-zona pellucida binding, acrosome reaction, and sperm DNA fragmentation) [17] are required to comprehensively evaluate fertilisation potential. Thus, findings based solely on conventional semen analysis must be interpreted with caution when inferring the true impact of SSBs on male fertility.

Fifthly, while some studies included participants from East Asian populations, the majority of cohorts were still predominantly Caucasian. Moreover, there remains a paucity of research focusing on the broader diversity within Asian populations, particularly South and Southeast Asian groups. Given that Asia is the largest and most populous continent, accounting for nearly 60% of the world’s population [51] (4.3 billion people, including Chinese, Indians, Japanese, Vietnamese, Koreans, Filipinos, Thai, Malaysians, Indonesians, and Singaporeans), this leaves a critical gap in understanding how dietary, environmental, and cultural differences may influence the above-mentioned associations. Consequently, the generalisability of findings from existing studies remains limited when applied across diverse Asian ethnicities.

Finally, considerable heterogeneity was observed across the studies in terms of exposure definitions, study designs, sample sizes, and outcome measures. These differences complicate the direct comparison of results and preclude meta-analytic pooling at this stage.

It is important to consider that incomplete adjustment for lifestyle and environmental exposures across studies may have influenced the observed associations, underscoring the complexity of disentangling diet-specific effects from broader health-related confounders.

### 4.7. Future Directions

To strengthen the current pool of evidence, longitudinal studies with standardised exposure measurements and semen analysis protocols are necessary. Incorporating biomarkers of oxidative stress and detailed dietary assessments could further enhance the mechanistic understanding of the impacts of SSBs. Moreover, research focusing on Asian populations would be particularly valuable, given the dietary and genetic variations that may influence susceptibility to SSB-induced reproductive changes. Further studies into particular subgroups of populations will better capture nuances in lifestyle and dietary habits that may also potentially impact male fertility. Additionally, future research should also incorporate broader exposome profiling, including sleep quality, pollutant exposure, and dietary antioxidant intake, to reduce residual confounding.

### 4.8. Recommendations

**Figure 1:**
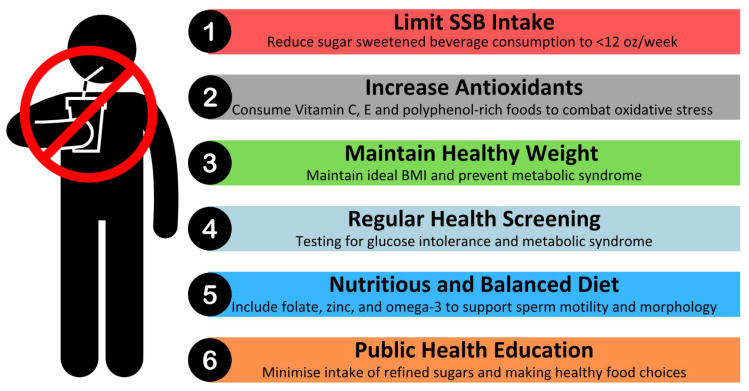
Recommendations for reducing the impact of SSBs on sperm health.

The current body of evidence suggests that the regular consumption of SSBs is associated with adverse effects on sperm health, including reductions in sperm count and motility, as well as increased DNA fragmentation. To mitigate these effects and promote better reproductive health, a combination of dietary modifications, lifestyle changes, and public health interventions is recommended. These practical recommendations are summarised in Figure 1 for ease of reference.

Limiting SSB consumption is a key strategy. The World Health Organisation recommends that both adults and children should reduce daily intake of free sugars to less than 10% of their total energy intake, with a reduction to below 5% (roughly 25 g or 6 teaspoons) per day providing additional health benefits [52]. One standard Coca Cola can (12 ounces) contains 39 g of sugar [53]. Therefore, it is advisable to limit intake of SSBs to less than 12 ounces per week.

In parallel, public health efforts should encourage individuals to opt for healthier alternatives such as water, herbal teas, or beverages with minimal or no added sugar. In countries such as Singapore, Nutri-Grade labelling classifies beverages based on sugar content. Consumers are encouraged to pick beverages labelled with Nutri-Grade “A”, which contain ≤1 g of sugar [54].

Beyond limiting SSB intake, dietary modifications play a significant role in sperm health. A nutrient-dense diet rich in antioxidants, essential minerals, and healthy fats can counteract oxidative stress and support sperm function [55]. Consuming foods high in vitamins C and E, zinc, folate, and omega-3 fatty acids has been shown to improve motility and reduce DNA damage. These dietary components help combat the oxidative stress triggered by high sugar intake, which is a key contributor to sperm dysfunction.

Maintaining a healthy weight and metabolic profile is another critical factor in preserving sperm health. Obesity and metabolic syndrome, both exacerbated by excessive sugar consumption, are associated with insulin resistance, hormonal imbalances, and inflammation, all of which negatively impact sperm parameters. We recommend that individuals maintain a healthy BMI through balanced nutrition and regular physical activity. Engaging in mild–moderate intensity physical exercises may support metabolic health and improve reproductive outcomes [56]. Given that SSB consumption has been linked to hormonal disruptions, regular monitoring of testosterone and insulin levels may be particularly beneficial for men experiencing fertility concerns. Routine health check-ups may help identify early indicators of metabolic syndrome, oxidative stress, or endocrine imbalances, and this would enable timely dietary and lifestyle interventions to mitigate potential reproductive complications.

From a broader public health perspective, policy interventions can play a significant role in reducing SSB-related health risks. Strategies such as sugar taxation, clearer food labelling, and educational campaigns have demonstrated effectiveness in reducing SSB consumption in various populations. Governments and health organisations should continue to advocate for such initiatives, promoting a shift toward healthier dietary patterns that benefit both overall health and reproductive function.

## 5. Conclusions

In conclusion, the existing body of literature suggests a potential link between the consumption of SSBs and male infertility, particularly through adverse effects on sperm parameters. Several studies indicate that the regular intake of SSBs, even in moderate amounts, may be associated with reduced sperm count, motility, and morphology. The high sugar content and the presence of additives, such as artificial sweeteners and preservatives, may contribute to oxidative stress, hormonal imbalances, and metabolic dysfunctions, all of which can impair spermatogenesis and sperm health. This will, in turn, affect male fertility and overall reproductive health, and the effects of metabolic dysfunction may even have a negative impact on overall health and longevity.

However, while these findings are compelling, they remain inconsistent across studies due to variations in study designs, population demographics, and methods of measuring SSB intake and sperm parameters. Further research is needed to establish a clearer causal relationship and to understand the mechanisms underlying these associations. Moreover, public health interventions aimed at reducing SSB consumption, particularly among young men, may help mitigate the potential reproductive challenges they may face as they reach child-bearing age. Specific research in other Asian populations, such as the South Asian and Southeast Asian cohorts, would also be crucial in identifying the nuances in SSBs and their effect on Asian male fertility, as current research has largely been focused on Caucasian and some East Asian cohorts.

Addressing SSB consumption as part of a broader lifestyle modification strategy could be an important consideration in the optimisation of male reproductive health, whereby semen quality predicts mortality risks in men [7]. Overall, while the current evidence suggests some association between SSB intake and sperm quality, definitive conclusions require larger, longitudinal studies with more controlled variables to strengthen our understanding of male reproductive health.

## Figures and Tables

**Table 1:** Characteristics of primary studies included.

Author (Year)	Study Period	Study Design	Population	Exposure (SSBs)	Outcome Measure	Key Findings	Limitations
Jensen et al. (2010) [24]	2001–2006	Cross-sectional Study	The Netherlands (*n* = 2554 males) Aged ≥ 18	Bottles of Coca Cola (0.5 L) in a week	Semen volume, sperm concentration, total sperm count, morphology	No association of coffee, tea, chocolate beverages, or diet soft drinks with semen quality Only Coca Cola consumption is associated with a significant reduction in semen quality Only Coca Cola affected semen quality—it could have been sugar in the Coca Cola affecting semen	The study was for caffeine exposure Found that a high-quantity consumers of Coca Cola/caffeine have unhealthy lifestyles, which may confound the results The study population mainly was Caucasian
Chiu et al. (2014) [25]	2009–2010	Cross-sectional Study	United States (*n* = 189 males) Aged 18–22	Carbonated SSBs with caffeine Carbonated SSBs without caffeine Non-carbonated SSBs Fruit juices Servings (12 oz)—never 6 or more servings a day	Sperm motility, sperm concentration, morphology, ejaculate volume, reproductive hormones	Intake of SSBs is related to lower sperm motility (total and progressive) Slightly lower FSH with higher SSB intake	Not possible to determine to what extent the observed relations with sperm motility might translate into fertility
Liu et al. (2015) [26]	2008–2013	Cross-sectional Study	Taiwan (*n* = 7282 males) Aged ≥ 18	Cake or cookies Additional sugars Sweetened beverages <1/week 1–3/week 4–6/week 1/day >2/day	Sperm concentration, total sperm motility, progressive motility, normal sperm morphology	Highly sweet snacks and sugar-sweetened drinks are associated with lower sperm concentration and a lower percentage of normal sperm morphology	No subdivision of sugar-sweetened drinks into separate categories Snacks and SSBs combined into one category
Yang et al. (2015) [27]	2013–2014	Cohort Study	China (*n* = 796 males) Median age = 20	Milk tea Coca Cola Never <3 cups/week ≥3 cups/week	Semen appearance, semen volume, sperm morphology, sperm concentration, sperm motility, serum sex hormones	Coca Cola consumption is associated with decreased semen volume	The population studied was mainly Han Chinese Studied only Coca Cola—contains caffeine which may be a confounder
Wesselink et al. (2016) [28]		Cohort Study	North America (*n* = 662) Aged ≥ 21	Caffeinated soda Energy drinks 0, <1, 1, >2 cans/day	Fecundability	Both soda and energy drink intake are associated with reduced fecundability	The study was conducted for caffeine Energy drinks were not separated into sugar vs. diet drinks
Hatch et al. (2018) [29]	2013–2017	Cohort Study	North America (*n* = 1045) Aged ≥ 21	Sugar-sweetened soda Diet soda Energy drinks Sweetened sports drinks Fruit juices No. of 12-ounce servings per week	Fecundability	SSB is associated with reduced fecundability Diet soda did not affect fecundability The largest reduction seen was in men who consumed 7 or more energy drinks per week	Lacked info on male dietary factors that can confound results—high sugar foods
Nassan et al. (2021) [30]	2008–2017	Cross-sectional Study	Netherlands (*n* = 2935 males) Median age = 19	Intake of SSBs assessed via a validated food frequency questionnaire (FFQ) The median SSB intake in the highest category was 1.1 servings/day (~220 mL/day)	Semen parameters: sperm concentration, total sperm count, motility, and morphology Serum reproductive hormones: inhibin-B, FSH, testosterone, and others	SSB consumption was associated with lower sperm concentration, lower total sperm count, and a reduced inhibin-B/FSH ratio (impaired spermatogenesis) Possible mechanisms: oxidative stress and disrupted hypothalamic–pituitary–gonadal axis due to insulin resistance and cellular aging effects	Cross-sectional design limits causal inference Self-reported SSB intake may introduce reporting biases A single semen sample per participant may not reflect long-term sperm quality
Efrat et al. (2022) [31]	2012–2015	Cross-sectional Study	Israel (*n*= 593 males) Aged 18–55 years	(1) Soft drink consumption (SoftD, 1 drink = 8 fl/oz) (2) Sugar content in consumed food products	Sperm parameters: volume, sperm concentration, total sperm count, percentage of motility, percentage of normal morphology	The sperm concentration and total sperm count are negatively associated with the consumption of SSBs Possible mechanisms: increased oxidative stress	Cross-sectional study design, unable to demonstrate cause and effect, and unable to completely rule out the possibility of residual confounders
Kiwitt-Cárdenas (2022) [32]	2010–2011	Cross-sectional Study	Spain (*n* = 209) Aged 18–23	The intake of SSBs assessed via a validated food frequency questionnaire (FFQ) Carbonated SSBs with caffeine Carbonated SSBs without caffeine Sugar-free carbonated SSBs Bottled fruit juice One serving = 330 mL	Ejaculate volume, sperm motility, sperm concentration, total sperm count, sperm morphology Reproductive hormones: LH, FSH, sex hormone-binding globulin, testosterone, inhibin B	Higher intake of SSB is associated with higher percentage of normal sperm morphology and increased serum levels of estradiol. No statistically significant associations with other semen parameters or other reproductive hormones	Cross-sectional study, unable to demonstrate cause and effect Only one sample per participant was analysed
Meldgaard et al. (2022) [33]	2017–2019	Cross-sectional Study	Netherlands (*n* = 5697 males) Aged 18	Sugar-sweetened and artificially sweetened beverages Moderate (≥3 days a week) and infrequent (<3 days a week) consumption	Sample volume, sperm concentration, total sperm count, total motility, morphology	Neither the consumption of sugar-sweetened nor artificially sweetened beverages was strongly associated with semen volume, sperm concentration, total motility, and total sperm count in this study	The cross-sectional study design was unable to demonstrate cause and effect Self-reported via recall of consumption, which may lead to measurement error of exposure
Joseph et al. (2024) [34]	2015–2022	Cohort Study	North America (*n* = 690 males) Aged ≥ 21	Sugar-sweetened soda, energy drinks, fruit juice, and sports drinks Calculated total SSB consumption by summing up weekly intake values of sugar-sweetened sodas, sugar-sweetened energy drinks, fruit juices, and sports drinks	Self-evaluation of semen quality using an at-home semen testing system Semen volume, sperm concentration, total sperm count, motility, total motile sperm count	SSB intake was inversely associated with all five semen parameters	Potential for exposure misclassification given that we evaluated SSB consumption at baseline and spermatogenesis takes approximately 74 days to occur Although adjusted for several covariates, unmeasured or residual confounding from lifestyle and behavioural factors (e.g., healthy diet) remains possible Measured semen quality using an at-home semen testing kit rather than the gold standard of laboratory assessment
